# Laparoscopic Cholecystectomy in Cirrhotic Patient

**DOI:** 10.1155/1996/67964

**Published:** 1996

**Authors:** Jean Gugenheim, Marco Casaccia, Davide Mazza, James Toouli, Vanna Laura, Pascal Fabiani, Jean Mouiel

**Affiliations:** 1 Department of Digestive Surgery Video-Surgery and Hepatic Transplantation St. Roch Hospital University of Nice-Sophia Antipolis France; 2 Flinders Medical Center Bedford Park Australia

## Abstract

Cholecystectomy is associated with increased risk in patients with liver cirrhosis. Moreover, cirrhosis and portal hypertension have been considered relative or absolute contraindication to laparoscopic cholecystectomy. As experience with laparoscopic cholecystectomy increased, we decided to treat cirrhotic patients via this approach. Between January 1994 and April 1995, nine patients with a Child-Pugh's stage A cirrhosis underwent elective laparoscopic cholecystectomy with intraoperative cholangiography. There was no significant per- or post-operative bleeding and no blood transfusion was necessary. There was no mortality and very low morbidity. Median hospital stay was 3 days. This series suggests that wellcompensated cirrhosis can not be considered a contraindication to laparoscopic cholecystectomy.


*HPB Surgery*, 1996, Vol.10, pp. 79–82
Reprints available directly from the publisher
Photocopying permitted by license only

© 1996 OPA (Overseas Publishers Association).
Amsterdam B.V. Published in The Netherlands
by Harwood Academic Publishers
Printed in Malaysia

### Laparoscopic Cholecystectomy in Cirrhotic Patient

JEAN GUGENHEIM*, MARCO CASACCIA JR.*, DAVIDE MAZZA*, JAMES TOOULI**, VANNA LAURA*, PASCAL FABIANI* and JEAN MOUIEL*

*Department of Digestive Surgery, Video-Surgery and Hepatic Transplantation St. Roch Hospital
University of Nice-Sophia Antipolis, France
**Flinders Medical Center, Bedford Park, South Australia

*(Received 25 October 1995)*

Cholecystectomy is associated with increased risk in patients with liver cirrhosis. Moreover, cirrhosis and portal hypertension have been considered relative or absolute contraindication to laparoscopic cholecystectomy. As experience with laparoscopic cholecystectomy increased, we decided to treat cirrhotic patients via this approach. Between January 1994 and April 1995, nine patients with a Child-Pugh's stage A cirrhosis underwent elective laparoscopic cholecystectomy with intraoperative cholangiography. There was no significant per- or post-operative bleeding and no blood transfusion was necessary. There was no mortality and very low morbidity. Median hospital stay was 3 days. This series suggests that well-compensated cirrhosis can not be considered a contraindication to laparoscopic cholecystectomy.

KEY WORDS: Cirrhosis laparoscopic cholecystectomy

### INTRODUCTION

Gallstones are more frequent in cirrhotic patients than in the general population[1,2]. Cholecystectomy is associated with increased risk in patients with cirrhosis because of intraoperative bleeding, postoperative hepatic failure and sepsis with the potential of multiple organ failure[3–8]. However at times, it becomes necessary to treat patients with cirrhosis who have gallstones disease as they present with debilitating symptoms[9]. Non cirrhotic patients with symptomatic gallstones currently are treated by Laparoscopic Cholecystectomy (LC). Cirrhosis and portal hypertension have been considered relative or absolute contraindication to LC[10–12]. However, as experience with LC increased, a number of cirrhotic patients have been treated via this approach. We report our experience of laparoscopic cholecystectomy in the cirrhotic patient.

*Correspondence to:* Prof. Jean Gugenheim, Department of Digestive Surgery, Video-Surgery and Hepatic Transplantation St. Roch Hospital-5, Rue Pierre Devoluy. 06000 Nice.

### MATERIALS AND METHODS

Between January 1994 and April 1995, 9 cirrhotic patients underwent laparoscopic cholecystectomy. Operations were performed by two surgeons (J.G., P.F.) with a well-established experience in laparoscopic surgery. There were 6 males and 3 females with a median age of 59 years (range: 35–81 years). Six patients had cirrhosis confirmed by liver biopsy; in the remaining three the diagnosis was made on the basis of significant clinical history and the appearence of the liver at operation. Cirrhosis was secondary to: post-hepatitis C in 4 cases, alcohol in 3 cases, post-hepatitis B and hemocromatosis. All patients were stage A according to the Child-Pugh classification. No patient had ascites preoperatively. Two had stage I and II esophageal varices. One of these two patients had a program of sclerotherapy before he became symptomatic. The other patient was operated on without sclerotherapy. One patient was HIV infected. All patients had preoperative and postoperative liver function tests (see Tab. 2) and abdominal ultrasonography.

**Table 1** Case reports

| | *case 1* | *case 2* | *case 3* | *case 4* | *case 5* | *case 6* | *case 7* | *case 8* | *case 9* |
|---|---|---|---|---|---|---|---|---|---|
| Age | 61 | 41 | 70 | 81 | 45 | 43 | 59 | 35 | 76 |
| Sex | F | F | M | M | M | M | M | M | F |
| Etiology of cirrhosis | hemocromatosis | alcohol | alcohol | post-hepatitis C | alcohol | post-hepatitis B | post-hepatitis C | post-hepatitis C | post-hepatitis C |
| Child's class | A | A | A | A | A | A | A | A | A |
| Symptoms | biliary colic | acute cholecystitis | biliary pancreatitis | biliary colic | acute cholecystitis | biliary colic | biliary colic | acute cholecystitis | biliary colic |
| Transfusion | - | - | - | - | - | - | - | - | - |
| Median operative time | 90' | 80' | 90' | 55' | 70' | 105' | 60' | 80' | 90' |
| Peroperative cholangiography | normal | normal | normal | normal | normal | normal | normal | normal | normal |
| Duration of hospital stay (days) | 7 | 3 | 2 | 5 | 3 | 3 | 3 | 2 | 4 |

**Table 2** Case reports - Liver function tests

| | *case 1* | *case 2* | *case 3* | *case 4* | *case 5* | *case 6* | *case 7* | *case 8* | *case 9* |
|---|---|---|---|---|---|---|---|---|---|
| AST | 44/52 | 46/79 | 40/41 | 238/381 | 125/86 | 75/44 | 266/261 | 171/138 | 37/73 |
| ALT | 37/39 | 55/80 | 48/46 | 216/319 | 159/165 | 86/63 | 269/274 | 88/71 | 42/61 |
| ALP | 129/99 | 98/88 | 102/80 | 194/207 | 102/75 | 96/61 | 178/98 | 391/414 | 82/55 |
| GGT | 48/38 | 120/102 | 135/109 | 323/329 | 119/77 | 194/162 | 231/220 | 930/1933 | 48/41 |
| T.Bil. | 11,9/16,5 | 9,6/15,0 | 0,0/0,0 | 40,2/84,7 | 17,6/19,5 | 13.1/17,7 | 16,8/26,8 | 80,7/74,6 | 17,2/26,5 |
| PT | 81/67 | 100/90 | 65/68 | 85/76 | 89/77 | 76/60 | 100/73 | 78/67 | 90/78 |
| Platelets | 141.000 | 200.000 | 152.000 | 193.000 | 76.000 | 60.000 | 75.300 | 134.000 | 186.000 |

AST, aspartate amino transferase (U.I.); ALT, alanine amino transferase (U.I.); ALP; alkaline phosphatase (U.I.); GGT, gamma glutamil transferase (U.I.); T.Bil., total bilirubin (mcmol/L); PT, prothrombin time (%); Platelets, n/mm$^3$. (preoperative values/postoperative values)

No patient had previous upper abdominal surgery. Indications for surgery were: recurrent biliary colic in 5 cases, acute cholecystitis in 3 cases and biliary pancreatitis in 1 case. In this latter case, a preoperative ERCP was carried out, and demonstrated the presence of micro-crystals in the bile. This patient had an alcoholic cirrhosis, but a complete withdrawal was advocated by the patient and his family. This led us to indicate a cholecystectomy in this patient. None of the patients were operated as an emergency; 2 had the operation during the same hospital admission for an acute episode and 7 had an elective operation at a later date. Surgery consisted of laparoscopic cholecystectomy and routine preoperative cholangiography, using our standard technique described previously[13]. Liver biopsy was done in three patients to confirm the etiology and the degree of cirrhosis. Abdominal drains were not used. Antibiotics were given for 48 hours and started at the induction of general anesthesia. They consisted of piperacilline I.V. 8 gr. per day in 5 patients, and of amoxicilline + clavulinic acid 3 gr. per day in 4 patients. After operation patients were followed up as outpatients by their surgeon at regular intervals.

### RESULTS

Cholecystectomy was completed laparoscopically in all of the nine patients. During the operation, we did not find any difficulty to dissect the triangle of Calot. However, the dissection of the gallbladder from the cirrhotic liver was difficult. After a moderate traction of the gallbladder, division was performed very cautiously using the diathermy hook. Haemostasis was completed carefully by use of a diathermy spatula, argon beam diathermy and fibrin glue (Tissucol, Immunofrance S. A., Illkirch, France). There was no significant peroperative or postoperative bleeding in any of the patients and no blood transfusion was necesssary. All peroperative cholangiograms were normal and showed no evidence of stones in the bile ducts. The gallstones were pigmented in all patients. In eight they were multiple and one patient had a solitary stone. Median duration of the procedure was 80 minutes (range: 55–105 min.). Postoperative course was uneventful in 8 cases, 1 patient had moderate ascites, which was sucessfully treated with diuretics and did not require paracentesis. There was no significant alteration of the liver function tests, and no sign of liver decompensation. There was no mortality. Median hospital stay was 3 days (range: 2–7 days). Median duration of follow-up was 12.5 months (range: 7–22 months). One patient (n°7) developed a multifocal hepatocellular carcinoma and died 10 months after the operation. All other patients are alive without any complication.

### DISCUSSION

There are a number of reports[3–5] which alert us to the potential morbidity (30–83%) and mortality (21–27%) associated with cholecystectomy in the cirrhotic patient. In these reports it is documented that the greater the severity of the cirrhosis, the greater is the potential of complications. In other series[3,7,14,15], similar to ours, cholecystectomy has been performed in cirrhotic patients without significant morbidity or mortality. The difference between the former and later reports is in the severity of cirrhosis. Our patients were well compensated and all of Child Pugh class A. In such patients, despite the difficulties presented by alteration in the anatomy of the liver, due to the cirrhosis, laparoscopic cholecystectomy is possible and is associated with little increase in the risk of complications. In addition, none of our patients needed to undergo emergency surgery, a factor that is considered[5,14] a poor predictor of outcome for a cirrhotic patient undergoing an abdominal operation.

Wound problems, such as infection, dehiscence and postoperative hernia, are complications which result in postoperative morbidity after open cholecystectomy in cirrhotic patients, with a reported incidence of 12%[8]. We had no wound complications following LC and we believe that this is a major advantage in treating these patients via this approach.

Potentially, laparoscopic surgery is associated with less adhesions around the liver, and there is no subcostal incision. These factors may be important if the patient were to need liver transplantation at a future date.

These cases were operated by experienced laparoscopic surgeons. We caution that a surgeon must possess a high degree of laparoscopic proficiency before performing laparoscopic cholecystectomy in the cirrhotic patient. We recommend a low threshold for conversion to the open procedure if difficulties, such as significant bleeding, are encountered.

However, our series has demonstrated that laparoscopic cholecystectomy is feasible in the well-compensated cirrhotic patient, with potential advantages when compared to the open procedure.

### REFERENCES

1. Bouchier, I.A.D. (1969)Postmortem study of the frequency of gallstones in patients with cirrhosis of the liver. *Gut.*, **10**, 705–10.
2. Nicholas, P., Rinaudo, P.A., Conn, H.O. (1972) Increased incidence of cholelitiasis on Laennec's cirrhosis. *Gastroenterology.*, **63**, 112–21.
3. Schwartz, S.I. (1981)Biliary tract surgery and cirrhosis. A critical combination. *Surgery.*, **90**, 577–83.
4. Aranha, G.V., Sontag, S.J., Greenle, H.B. (1083) Cholecystectomy in cirrhotic patients. A formidable operation. *Am J Surg.*, **143**, 306–9.
5. Garrison, R.N., Cryer, H.M., Howard, D.A., Polk, H.C. (1984) Clarification of risk factors for abdominal operations in patients with hepatic cirrhosis. *Ann Surg.*, **199**, 648–55.
6. Doberneck, R.C., Sterling, N.A., Allison, D.C. (1983) Morbidity and mortality after operation in nonbleeding cirrhotic patients. *Am J Surg.*, **146**, 306–9.
7. Kogut, K., Aragoni, T., Ackermann, N.B. (1985) Cholecytectomy in patients with mild cirrhosis. A more favourable situation. *Arch Surg.*, **120**, 1310–1.
8. Bloch, R.S., Allaben, R.D., Walt, A.J. (1985) Cholecystectomy in patients with cirrhosis. A surgical challenge. *Arch Surg.*, **120**, 669–72.
9. Gillet, M. Chirurgie des voies biliaires chez le cirrhotique. AFC
10. Zucker, K.A., Bailey, R.W., Gadacz, T.R. *et al.* (1991) Laparoscopic guided cholecystectomy: a plea for cautious enthusiasm. *Am J Surg.*, **161**, 36–44.
11. Cuschieri, A., Dubois, F., Mouiel, J. *et al.* (1991) The European experience with laparoscopic cholecystectomy. *Am J Surg.*, **161**, 385.
12. Gadacz, T.R., Talamini, M.A. (1991) Traditional versus laparoscopic cholecystectomy. *Am J Surg.*, **161**, 336–8.
13. Fabiani, P., Iovine, L., Kathkouda, N., Gugenheim, J., Mouiel, J. (1993) Dissection du triangle de Calot par voie coelioscopique. *Presse Med.*, **22**, 535–537.
14. Wong, R., Rappaport, W., Witte, C. *et al.* (1994) Risk of nonshunt abdominal operation in the patient with cirrhosis. *J Am Coll Surg.*, **179**, 412–416.
15. Vanlandingham, S.B., (1984) Cholecystectomy in cirrhotic patients. *South Med J.*, **77** 38–40.

### COMMENTARY

### Laparoscopic Surgery: A Super-Speciality?

This paper by Gugenheim *et al.* highlights a growing trend in minimally invasive surgery. This trend recognises that there are groups of high risk patients who will significantly benefit from a minimally invasive approach. These include the morbidly obese, patients who have had multiple previous laparotomies (Crohn's disease) and patients where there is a likelihood of major surgery in the future, such as liver transplantation for those with cirrhosis. All these, by avoiding the higher morbidity of open surgery or the reduction in adhesion formation, are offered a benefit. To datethese situations have been seen as relative or absolute contraindications to minimally invasive surgery because of technical difficulties. It is becoming clear and highlighted again by Gugenheim *et al.* that with growing expertise, minimally invasive operations are achievable with good sucess rates in such groups.

The published literature demonstrates that cirrhotic patients have a much higher morbidity rate with open cholecystectomy, the desire to leave the right upper quandrant relatively adhesion free, should a need for a liver transplantation arise, is understandable.

The benefit offered to the cirrhotics described is achieved because such patients are treated within the confines of a specialist hepato-biliary unit and a unit with expertise in minimally invasive surgery.

The risks of submitting patients with symptomatic gallstones to an open operation, known to have higher morbidity because of their underlying medical condition, can now be obviated with confidence. However, the follow on from this is that there must be recognition within the surgical community that there are surgeons who have accumulated or are rapidly accumulating the necessary experience to achieve these consistent results. Thus the temptation to subject high risk groups of patients to elective open operations must be resisted and referral to specialist surgeons sought.

P. Declan Carey MCh FRCSI
Consultant Surgeon
Department of Surgery
University of Wales College of Medicine
Heath Park
Cardiff CF4 4XN

